# All-on-Four Rehabilitation: A Systematic Review of Clinical Management, Workflow and Complications

**DOI:** 10.3390/dj14090541

**Published:** 2026-08-31

**Authors:** Benjamin Reto Huber, Noah Walser, Thiemo Gamma, Philipp Metzler

**Affiliations:** 1 Oral- and Cranio-Maxillofacial Surgery, Cantonal Hospital Aarau, Tellstrasse, 5001 Aarau, Switzerland; 2 Klinik für Zahnerhaltung und Präventivzahnmedizin, Bereich für Parodontologie & Periimplantäre Erkrankungen, Zentrum für Zahnmedizin der Universität Zürich, Plattenstrasse 11, 8032 Zurich, Switzerland; 3 Clinic of Cranio-Maxillofacial and Oral Surgery, Center of Dental Medicine, University of Zurich, Plattenstrasse 11, 8032 Zurich, Switzerland

**Keywords:** all-on four, all-on 4 fixed dental prosthesis, tilted implants, immediate loading

## Abstract

**Background:** Severe tooth loss affects approximately 267 million people worldwide and has a significant impact on quality of life and general health. Various prosthetic treatment concepts exist for oral rehabilitation. The All-on-Four concept, first described by Maló in 2003, is considered a modern approach for timely and patient-friendly restoration of oral function and esthetics. **Methods:** A systematic literature search was conducted in the electronic databases MEDLINE/PubMed and EMBASE (Elsevier). Eligible studies were identified according to the framework, including investigations on the All-on-Four concept (PIO) and alternative implant-supported full-arch rehabilitation strategies (FDP4+, PCO). The methodological quality of the included studies was assessed using the Newcastle–Ottawa Scale. **Results:** Following the selection process, eleven PIO studies and nine PCO studies were included for qualitative analysis. Reported prosthesis survival rates were comparable between All-on-Four (85.0–100%) and concepts with more than four implants (88.2–100%) over up to 20 years. Implant survival ranged from 89.7–97.9% for All-on-Four and 98.9–99.0% for alternative concepts. Marginal bone loss after 5 years reached up to 1.9 mm for All-on-Four and 2.9 mm for alternative concepts. Metal–ceramic prostheses demonstrated lower fracture rates (7.27–18.2%) compared with metal–acrylic restorations (7.3–36.7%). **Discussion:** The available evidence suggests that the All-on-Four concept represents a predictable treatment option for edentulous patients, with implant and prosthetic survival rates comparable to FDP4+ concepts. Immediate loading protocols may improve patient satisfaction and reduce treatment time when adequate primary stability is achieved. However, biological and technical complications remain clinically relevant. Interpretation of the findings is limited by heterogeneous study designs and predominantly retrospective evidence. **Conclusions:** The All-on-Four concept provides a reliable rehabilitation option for patients with advanced ridge atrophy who decline regenerative procedures or removable prostheses. Current evidence indicates no significant differences in survival or complication rates compared to other implant-supported treatment concepts.

## 1. Introduction

Severe tooth loss up to complete edentulism affects approximately 267 million individuals worldwide [1]. The resulting impairment of mastication, aesthetics, and phonetics, combined with reduced self-esteem and associated social limitations, leads to a significant decrease in quality of life [2].

Oral rehabilitation therefore plays a crucial role in restoring patients’ former lifestyle and substantially improving their quality of life [3]. For completely edentulous patients, several prosthetic treatment approaches are available, including conventional complete dentures, removable implant-supported prostheses, and fixed implant-supported prostheses with more than four implants (FDP4+). Compared to oral rehabilitations without implants, FDP4+ results in improved denture stability and masticatory performance, while also reducing the load on the alveolar ridge and enhancing patients’ quality of life [4,5]. Nevertheless, rehabilitation with FDP4+ may, depending on the clinical situation, necessitate bone augmentation procedures (e.g., grafting or sinus elevation), multiple surgical interventions, and prolonged treatment duration. Such requirements can reduce patient acceptance and lead to a preference for less invasive non-implant solutions. Accordingly, there remains an unmet clinical need for innovative fixed implant-supported prostheses that minimize surgical complexity while improving patient acceptance.

Such a concept is represented by the All-on-Four approach (Nobel Biocare^®^, Zurich, Switzerland), which was first described by Maló et al. in 2003 [6]. This concept of fixed implant-supported rehabilitation, commonly known as the All-on-Four approach, is based on a prosthesis supported by four implants. In cases with limited bone availability, particularly posterior to the premolar region, this approach offers the advantage of avoiding extensive augmentation procedures. Following extraction of non-restorable teeth, four implants are placed immediately (two anterior and two posteriors, axially or tilted), and a provisional prosthesis of 10–14 teeth made of polymethylmethacrylate (PMMA) is delivered either on the same day or shortly thereafter, allowing early functional loading. After approximately six months of healing to ensure complete osseointegration and soft tissue maturation, the definitive prosthesis can be delivered [7,8,9].

For patients seeking a fixed full-arch prosthesis with a minimal number of implants, no augmentation procedures, and rapid rehabilitation, such a concept represents a suitable option. The Association of the Scientific Medical Societies (AWMF) S3 guideline does not recommend routine placement of more than four implants in edentulous jaws when adequate stability and prosthetic support can be achieved [10]. The reduced number of implants and the possibility of performing the procedure on an outpatient basis help to lower treatment costs [11,12].

The aim of this systematic review was to address the following focused question: in adult patients with edentulous jaws undergoing fixed implant-supported full-arch rehabilitation (P), how does the All-on-Four concept (I) compare with fixed implant-supported prostheses on more than four implants (FDP4+) (C) with respect to implant survival, prosthesis survival, marginal bone loss, and biological and technical complications (O)? Key aspects considered included patient selection, clinical indications, surgical execution, as well as the implant and prosthetic materials used, and their impact on both mechanical and biological complications. The results are discussed in comparison with other fixed implant-supported rehabilitations involving more than four implants per arch.

## 2. Materials and Methods

### 2.1. Protocol and Reporting Guidelines

This systematic review was conducted in accordance with the recommendations of the PRISMA 2020 Statement (Preferred Reporting Items for Systematic Reviews and Meta-Analyses) [13]. The completed PRISMA checklist is provided in the Supplementary Material, with a link to the checklist available therein. The methodology and reporting were designed to ensure transparency and reproducibility of the literature search, study selection, and data analysis. The review protocol was registered in the PROSPERO international prospective register of systematic reviews (registration number CRD420261352454).

For transparency, amendments made between the initial registration and the final review methodology are described below. At the start of the review (31 October 2025), the information sources were restricted to MEDLINE/PubMed and EMBASE, although additional databases, including Scopus and the Cochrane Library, had been considered in the initial protocol. The search terms and database-specific search strings were also refined to improve sensitivity and alignment with the eligibility criteria. The final search strategies are provided in Section 2.3.

Although quantitative meta-analysis was initially planned, a narrative synthesis was adopted on 25 March 2026, after full-text screening and data extraction. This amendment was made because substantial clinical and methodological heterogeneity in prosthetic materials, implant loading protocols, follow-up periods, and definitions of technical and biological complications precluded meaningful statistical pooling.

The review scope was also clarified during subsequent protocol updates. Earlier PROSPERO entries contained inconsistencies regarding a strict mandibular focus and/or a specific comparison of digital workflows. These entries were corrected to reflect the scope of the review as conducted. The final review included both maxillary and mandibular full-arch reconstructions, while a separate comparison of digital workflows was not performed because sufficient eligible long-term comparative evidence was unavailable. The predefined outcome domains remained unchanged; however, some outcome definitions were refined during subsequent protocol updates to improve consistency and alignment with the methodology applied in the final review.

Secondary systematic reviews were excluded from the evidence synthesis and were used only as supplementary background sources. Overall, the amendments comprised refinements to the information sources and search strategy, the synthesis approach, and the clarification of the review scope and outcome definitions. The PROSPERO record was subsequently updated to ensure consistency between the registered protocol and the methodology reported in the completed review.

### 2.2. Eligibility Criteria and Search Terms

The eligibility criteria were defined according to the PICO framework [14]. The population consisted of adult patients (≥18 years) with edentulous jaws undergoing implant-supported full-arch rehabilitation. The search terms applied in MEDLINE/PubMed and EMBASE (Elsevier) were structured according to the PIO and PCO components (Table 1) The search was limited to publications written in English or German and published between January 2000 and January 2026, applying a “Title/Abstract” field filter. Both databases were last searched on 17 March 2026. The intervention of interest was the All-on-Four concept, while the comparison included alternative fixed implant-supported rehabilitations, such as All-on-Six or other fixed full-arch implant prostheses.

Studies were considered eligible if they evaluated implant-supported full-arch rehabilitations in edentulous jaws and were designed as clinical studies. Only studies including adult patients with a minimum follow-up period of four years and reporting surgical outcomes, prosthetic outcomes, implant survival rates, or biological and technical complications were included.

Studies involving partial prostheses, removable or hybrid removable prostheses, pediatric populations (<18 years), preclinical or in vitro investigations, conference abstracts, pilot studies, and systematic reviews were excluded. Only primary clinical studies were eligible for inclusion in the qualitative synthesis; systematic reviews and meta-analyses were not included as units of analysis, and the review was therefore conducted as a review of primary studies. In addition, studies involving regenerative procedures were excluded to improve comparability between treatment approaches.

### 2.3. Information Sources and Search Strategy

Two independent reviewers performed a systematic literature search in the electronic databases MEDLINE/PubMed and EMBASE (Elsevier). The search strategy combined Medical Subject Headings (MeSH) and free-text terms related to edentulous jaws, implant-supported full-arch rehabilitations, implant survival, and biological or technical complications. The full search strategies are presented in Table 1.

To ensure comprehensive identification of relevant studies, the electronic database search was supplemented by manual screening of the reference lists of included articles and relevant review papers. In addition, clinical guidelines from dental professional societies (notably the AWMF S3 guidelines), as well as relevant supporting literature, were screened for the Discussion Section.

Title and abstract screening and subsequent full-text assessment were carried out independently by two reviewers (B.R.H. and N.W.), who applied the predefined eligibility criteria without knowledge of one another’s decisions. Disagreements at either stage were first discussed between the two reviewers and, where consensus could not be reached, resolved by a third reviewer (P.M.) acting as adjudicator. The same independent, duplicate procedure was used for data extraction and for the risk-of-bias assessment.

### 2.4. Selection Process

**Figure 1:**
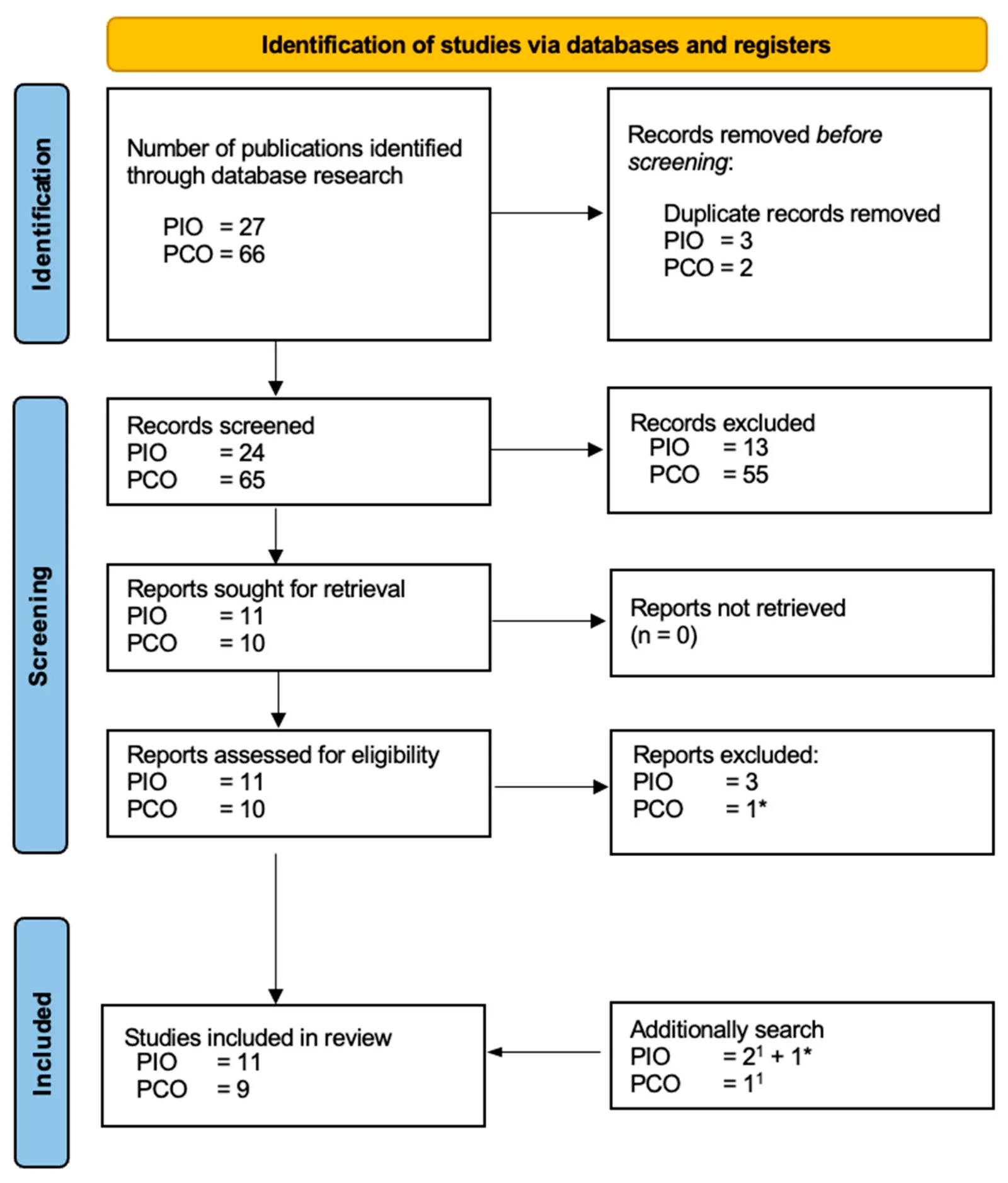
Prisma Flowchart for study selection: Literature selection process. * Study from PCO with an All-on-Four Concept (included in the PIO). ^1^ Manual search.

All identified records were imported into reference management software for further processing. Reference management and organization of the literature search were performed using Mendeley (Version 2.84.0). Duplicate records were removed prior to screening. Subsequently, titles and abstracts were screened according to the predefined eligibility criteria. Potentially relevant articles were then assessed through full-text review to determine their eligibility for inclusion in the review. The study selection process was documented using a PRISMA flow diagram, illustrating the number of records identified, screened, excluded, and included in the final analysis (Figure 1).

### 2.5. Data Extraction

The primary outcomes were defined as implant survival and prosthesis (reconstruction) survival. The secondary outcomes were marginal bone loss, biological complications (e.g., peri-implant mucositis and peri-implantitis), and technical complications (e.g., veneer chipping and prosthetic fracture). These outcomes were assessed separately for the All-on-Four concept and for FDP4+ rehabilitations to allow direct comparison between the two strategies. Due to the heterogeneity of study designs, outcome measures, and follow-up periods, a qualitative synthesis of the results was performed. The findings of the included studies were summarized descriptively. The following data were extracted from each study:

- Author and year of publication;
- Study design;
- Number of patients and implants;
- Type of implant-supported rehabilitation;
- Follow-up duration;
- Implant survival rates;
- Biological complications (e.g., peri-implantitis, MBL);
- Technical complications (e.g., chipping or prosthetic failures).

To ensure a methodologically consistent data analysis, the effect measures were defined a priori. Dichotomous outcomes were analysed using odds ratios (ORs), whereas continuous outcomes, such as marginal bone loss, were assessed using mean differences. Statistical significance was established when the corresponding parameters demonstrated significant effects, defined as a 95% confidence interval excluding the null value and a *p*-value < 0.05.

The characteristics and outcomes of the included studies were summarized in tables, including study design, patient characteristics, intervention and comparator characteristics, follow-up duration, and reported clinical outcomes.

### 2.6. Risk of Bias Assessment

The methodological quality of the included studies was assessed using the Newcastle–Ottawa Scale [15], which evaluates non-randomized studies across three domains: selection of study groups, comparability of cohorts, and assessment of outcomes. A maximum of nine stars could be awarded: up to four stars for selection, two for comparability, and three for outcome assessment. Studies scoring 7–9 stars were considered to have a low risk of bias, those with 4–6 stars a moderate risk, and those with 0–3 stars a high risk of bias. The assessment considered factors such as adequacy of study design, cohort comparability, duration and completeness of follow-up, ascertainment of outcomes, and transparency of methodological reporting. The certainty of the body of evidence was not formally assessed using GRADE or another established framework.

## 3. Results

### 3.1. Study Selection

The systematic search identified 27 publications related to the All-on-Four concept (PIO) and 66 publications related to alternative rehabilitation approaches (PCO). After removal of five duplicate records, titles and abstracts were screened for relevance. Following this screening step, 21 publications were assessed through full-text review (Table 2).

Four studies did not meet the inclusion criteria and were excluded after full-text assessment (Table 3). Consequently, nine publications addressing the All-on-Four concept and eight publications addressing alternative rehabilitation approaches were included in the qualitative analysis. In addition, two further publications by Hopp et al. and Tallarico et al., 2016 were identified through systematic citation tracking of the included studies [16,17]. This resulted in a total of 20 studies included in the final analysis.

Two studies conducted by La Monaca et al. and Toia et al. compared the All-on-4 concept with All-on-6 rehabilitations [18,19]. In this study, the results of the different rehabilitation approaches were analysed separately in order to allow for appropriate comparison between treatment modalities. The entire study selection process is illustrated in the PRISMA flow diagram (Figure 1).

### 3.2. Study Characteristics

The included studies investigated different aspects of implant-supported full-arch rehabilitation in edentulous jaws, with a particular focus on the All-on-Four concept and fixed implant-supported prostheses with more than four implants (FDP+4) concepts.

Across the included studies, the evaluated outcomes included patient selection criteria, clinical indications, surgical procedures, prosthetic rehabilitation protocols, implant survival rates, and biological and mechanical complications. The studies differed with respect to study design, follow-up duration, treatment protocols, and patient characteristics.

The risk of bias assessment using the Newcastle–Ottawa Scale demonstrated that the majority of included studies presented a moderate and low risk of bias. This was mainly attributable to their retrospective design, limited comparability between study groups, and absence of randomized controlled conditions.

A smaller number of studies were classified as having a low risk of bias, particularly those with prospective designs, clearly defined patient cohorts, and longer follow-up periods.

No study met the criteria for high risk according to NOS scoring; however, several limitations related to retrospective design and lack of comparability remained. Overall, the methodological quality of the included studies can be considered low-moderate, which should be taken into account when interpreting the results (Table 2).

### 3.3. Patient: Indications and Contraindication for All-on-Four

The indication for All-on-Four treatment in the included studies was primarily the rehabilitation of completely edentulous maxillae and/or mandibles in patients seeking a fixed implant-supported prosthetic solution [12,17,19,20,21,22,23]. In addition, several studies included patients presenting with terminal dentition, severe periodontal disease, extensive caries, or advanced bone resorption that compromised the prognosis of conventional restorative treatment [17,19,20,24]. Eligibility criteria commonly required patients to be in good general physical and mental health and able to undergo surgical treatment and adhere to postoperative maintenance protocols. Adequate oral hygiene and patient compliance with follow-up visits were also frequently reported as prerequisites for treatment inclusion [9,12]. Common contraindications for All-on-Four treatment included heavy smoking (>15 cigarettes/day), pregnancy or lactation, poor patient compliance, and insufficient bone volume preventing adequate implant stabilization [12,16,18,20,21,22,24]. Furthermore, patients with metabolic bone diseases, a history of bisphosphonate therapy, or previous radiotherapy or chemotherapy in the head and neck region were frequently excluded due to the increased risk of impaired osseointegration and postoperative complications [11,12,16,17,18,20,21].

### 3.4. Bone: Quantity and Quality

Adequate bone availability was commonly reported as an inclusion criterion, requiring sufficient bone volume and density in the anterior jaw region extending to the first or second premolar area. Specifically, a minimum horizontal bone width of >4 mm and a vertical bone height of >8 mm were considered necessary to achieve proper implant placement and primary stability. According to Lopes et al., patients presenting with a horizontal bone volume of >4 mm and a vertical bone height of >8 mm in the anterior region and up to the first or second premolars are suitable candidates for the All-on-Four concept (see Table 4) [22,24]. Furthermore, anterior bone availability extending to the second premolar region was considered sufficient for implant placement within the All-on-Four treatment protocol.

### 3.5. Surgical Procedure: Implant Insertion and Implant Type

Several studies reported immediate implant placement in All-on-Four rehabilitations, with five-year implant survival rates ranging from 94.5% to 99.6% and ten-year survival rates between 89.7% and 97.9% (see Table 5) [9,11,12,16,17,19,20,22,24]. Alternative fixed implant-supported prostheses with more than four implants (FDP4+) placed using delayed implantation protocols showed five-year survival rates ranging from 89.7% to 95.1% [25,26]. Five FDP4+ rehabilitations were performed using immediate implant placement protocols [19,27,28,29,30], reporting implant survival rates of 96.3–97.9% after one year and 94.4–96.1% after five years.

Most included studies investigating the All-on-Four concept applied immediate loading protocols, with the exception of Toia et al. (2025) and partial application in Tallarico et al. (see Table 5) [18]. Five-year implant survival rates associated with immediate loading ranged from 94.5% to 99.6% [9,16,17,20,21,22,23,24]. Three studies evaluating FDP4+ restorations reported five-year implant survival rates ranging from 89.7% to 100% [18,25,31] FDP4+ rehabilitations with immediate loading reported implant survival rates ranging from 94.4% to 96.3% after five years [28,30]. Implant diameters (3.2–5.0 mm vs. 3.25–5.5 mm) and implant lengths (7–18 mm in both groups) were comparable between the All-on-Four concept and FDP4+ configurations (see Table 3).

Self-tapping implants were used in several studies involving both the All-on-Four concept and FDP4+ restorations [11,12,16,19,20,21,22,23,24,27,28]. Implant survival rates for self-tapping implants ranged from 95.4% to 100% after seven years and from 89.7% to 100% after ten years (see Table 5). Bone-level implants demonstrated five-year survival rates between 89.7% and 99.6% [11,12,16,18,19,20,21,22,23,24,25,27,28,30]. Tissue-level implants showed five-year survival rates ranging from 96.2% to 100%, seven-year survival rates between 95.4% and 100%, and ten-year survival rates from 94.9% to 100% [9,12,16,17,18,20,22,23,25,26,27,28,30,31].

Distal implants in All-on-Four rehabilitations were most frequently positioned in the second premolar region, with insertion angles ranging from 30° to 45° [9,11,12,20,24]. No significant differences were reported between axial and tilted implants regarding implant survival rates or marginal bone loss (MBL) [17,19].

Most studies investigating the All-on-Four concept reported implant placement with a minimum insertion torque of 30 Ncm [9,11,17,20], whereas two studies reported a minimum insertion torque of 35 Ncm [12,16].

Alternative FDP4+ concepts involved the placement of additional implants distributed more extensively throughout the jaw [18,19,25,26,27,28,29,30,31]. Implant survival rates for the All-on-Four concept ranged from 95.5% to 100% after one year and from 94.5% to 100% after five years. FDP4+ rehabilitations reported implant survival rates above 96.3% after one year and above 94.4% after five years (see Table 5). Two long-term studies reported lower survival rates for restorations supported by four implants (90% after 25 years) compared with restorations supported by five or six implants, which showed survival rates of 98.9% after 20 years [21,31].

### 3.6. Prosthetic Procedure

Prosthesis survival for the All-on-Four concepts (85.0–100%) and FDP4+ restorations (88.2–100%) was comparable over similar follow-up periods (see Table 6). The most commonly fabricated temporary prostheses are made from PMMA. These prostheses exhibited a wide range of mechanical complications, ranging from 9.1% to 81.9%. Prosthesis survival for the All-on-Four concept ranged from 85.0% to 100%, while PMMA provisional prostheses in FDP4+ restorations demonstrated 100% survival (see Table 6). Most definitive prostheses were fabricated with a titanium framework and PMMA veneering. These metal–PMMA prostheses exhibited fracture rates ranging from 7.3% to 36.7% over a seven- to eleven-year follow-up period [24,32]. Instead of titanium–PMMA prostheses, alternative materials such as ceramic or zirconium dioxide can be used. Several studies reported metal–ceramic prostheses, which exhibited lower fracture rates compared to metal–PMMA restorations (7.27–18.20%) [9,11,12,17,19,20,22,24,26,28,31,32]. Metal–zirconia prostheses demonstrated fracture rates ranging from 5.36% to 29.7% [24,27]. Instead of a titanium framework, a zirconia framework can also be employed. Zirconia prostheses with ceramic veneering were reported in only one study [16]. No technical complications were reported for these zirconia prostheses.

### 3.7. Biological Complication

The reported biological complications varied considerably among the included studies and were inconsistently documented across follow-up periods. Marginal bone loss (MBL) generally remained within clinically acceptable ranges for both All-on-Four and FDP4+. In the All-on-Four group, reported MBL values after five years ranged from approximately 1.18 mm to 1.9 mm [9,11,19,24], while studies with follow-up periods exceeding seven years demonstrated MBL values between 1.3 mm and 2.5 mm [11,16] and 1.67 mm to 1.98 mm after ten years [9,20]. Studies evaluating FDP4+ demonstrated comparable biological outcomes. Reported MBL values in these studies generally ranged between 1.18 mm to 1.60 mm after five years follow-up [19,30,32]. Long-term data by Maló et al. demonstrated an MBL of 2.23 mm after 15 years for the All-on-Four concept, while FDP4+ restorations showed comparable outcomes, with an MBL of 1.4 mm after 14 years [20,31]. Long-term studies have reported MBL values ranging from 1.67 mm to 2.5 mm after ten years under immediate loading conditions [9,11,20]. By comparison, conventional loading protocols (healing period > 2 months) demonstrated an MBL of 1.4 mm after 15 years [31]. In three studies, marginal bone loss was nearly the same for axial implants (1.0 mm to 1.34 mm after five years) compared to angulated implants (1.10 mm to 1.27 mm after five years) [12,17,22].

The included studies reported varying results regarding biological complications such as implant infections, gingival inflammation, and peri-implant pathologies. Implant infections were documented in several studies, ranging from no cases to 203 affected patients. The highest number of implant infections was reported by Maló et al. (2019) with 203 cases among 1072 patients, while other studies described only isolated infections (e.g., Li et al., 2017; Cavalli et al., 2016) [9,12,23]. Several studies provided no data on this (see Table 7). Gingival inflammation was reported less frequently. The reported case numbers ranged from 0 to 189 patients. The highest prevalence was described by Hopp et al. (2017) with 189 cases, while other studies documented only low numbers or no inflammation [16,17,18,23]. In some studies, gingival inflammation was not reported at all. Peri-implant pathologies were also reported inconsistently. The number of affected patients ranged from 0 to 108 cases. The highest numbers were observed by Maló et al. (2019) and Hopp et al. (2017) with 108, 106 and 95 cases, respectively [9,17,20]. Other studies reported few or no peri-implant complications [18,19,24]. Overall, there was considerable variability among the studies regarding the recording and definition of biological complications, which limits direct comparability.

## 4. Discussion

### 4.1. Patient Indications and Contraindications for the All-on-Four Concept

Selection of the appropriate treatment concept for fixed implant-supported rehabilitation should be based on patient-related factors, anatomical conditions, and the available evidence (Table 4). The included studies suggest that fixed implant-supported prostheses may provide improved patient-reported satisfaction and oral health-related quality of life compared with removable prostheses in appropriately selected patients [33]. Consequently, patient preference for a fixed restoration represents an important consideration during treatment planning. However, the indication for an All-on-Four rehabilitation should always be balanced against anatomical limitations and patient-specific risk factors.

One of the principal indications for the All-on-Four concept is the presence of insufficient posterior bone volume that would otherwise require extensive augmentation procedures before placement of a greater number of implants. Vertical augmentation using autologous bone is associated with considerable morbidity, including graft exposure rates ranging from 12.5% to 33.3% and graft loss rates of 8–20% [34,35]. By utilizing tilted posterior implants and the available anterior bone, the All-on-Four concept may reduce the indication for complex grafting procedures while providing a fixed rehabilitation.

Patient age should also be considered when selecting the appropriate treatment strategy. According to the German AWMF S3 guideline, children and adolescents with anodontia should initially receive removable prosthetic rehabilitation until skeletal growth has been completed, whereas implant-supported fixed prostheses are recommended only after completion of craniofacial growth [36].

The available evidence further indicates that systemic conditions substantially influence implant treatment outcomes. Available evidence suggests that controlled systemic diseases, including diabetes and cardiovascular conditions, do not necessarily contraindicate implant therapy, although evidence for specific systemic conditions remains limited. In contrast, patients with previous head and neck radiotherapy, bone metastases, or high-dose antiresorptive therapy are associated with an increased risk of osteoradionecrosis or medication-related osteonecrosis of the jaw [37]. These patient groups were excluded from several studies evaluating the All-on-Four concept [11,12], thereby limiting the available evidence. Consistent with current AWMF recommendations, immediate loading protocols should generally be avoided or carefully considered in these high-risk patients [36]. Likewise, although implant therapy may improve prosthesis-related function in oncology patients, current guidelines generally recommend delayed implant placement with adequate implant distribution. Consequently, treatment concepts involving delayed loading, additional implants, or removable prostheses may be more appropriate in selected high-risk patients.

Behavioural factors should also be considered during patient selection. Long-term success is particularly dependent on patient adherence to supportive peri-implant maintenance. This consideration may be particularly relevant for full-arch fixed prostheses, where hygiene access may be more demanding compared with removable alternatives. Individuals with poor compliance, emotional instability, inadequate oral hygiene, or pregnancy were frequently excluded from the available studies, limiting the generalizability of current evidence [9,11,12,16,20,22,24]. Poor oral hygiene represents an established risk factor for peri-implant diseases, whereas elective implant placement is generally contraindicated during pregnancy because radiographic imaging and surgical intervention should be postponed whenever possible. Furthermore, periodontitis, uncontrolled diabetes (OR 1.65), smoking (OR 1.65–3.03), and bruxism (OR 2.71–8.70) have consistently been associated with increased risks of implant failure as well as biological and technical complications [9,20,22,29]. In accordance with current S3 guidelines, immediate or early loading protocols should therefore be carefully evaluated in patients with uncontrolled diabetes because impaired osseointegration may compromise treatment success [38]. Overall, the available evidence suggests that successful All-on-Four rehabilitation depends on careful patient selection. Comprehensive preoperative assessment, including evaluation of systemic health, behavioural risk factors, oral hygiene, smoking status, and anatomical conditions, together with appropriate risk-reduction strategies, is essential to minimize complications and optimize long-term clinical outcomes.

### 4.2. Bone Quantity and Quality

Edentulous patients frequently exhibit pronounced resorption of the posterior maxilla and mandible, which may preclude placement of implants with sufficient length and diameter using conventional rehabilitation concepts [28]. Consequently, extensive bone augmentation procedures are often required. Available observational evidence indicates that the All-on-Four concept may represent a predictable alternative in these situations by utilizing tilted posterior implants to maximize the available anterior bone and thereby reducing the need for grafting procedures.

Bone quantity appears to be an important determinant of long-term treatment outcomes. However, bone quantity should not be evaluated independently from bone quality, implant distribution, primary stability, and prosthetic biomechanics. Maló et al. demonstrated that greater residual alveolar bone volume was associated with improved implant survival and fewer mechanical complications. After five years, the highest implant survival rate (92.47%) was observed in patients with bone extending to the first premolar region, while bone extending to the second premolar significantly reduced the risk of mechanical complications (OR 0.22) [28]. These findings emphasize the importance of comprehensive preoperative assessment of both bone quantity and anatomical limitations during treatment planning.

Several studies also highlighted the importance of adequate ridge dimensions for achieving primary stability. To obtain a horizontal bone width exceeding 4 mm, vertical reduction of the alveolar ridge may occasionally be required, while preserving a minimum residual bone height of at least 8 mm to maintain favourable biomechanical conditions [22]. Overall, the available evidence indicates that the All-on-Four concept represents a valuable treatment option for patients with advanced posterior alveolar bone loss. Compared with conventional implant rehabilitation requiring extensive augmentation procedures, this approach may reduce surgical morbidity, treatment duration, and overall treatment costs while achieving comparable clinical outcomes in appropriately selected patients.

### 4.3. Surgical Procedure

One of the principal advantages of the All-on-Four concept is the possibility of immediate implant placement and immediate loading, allowing patients to regain oral function without a prolonged period of edentulism. The included studies generally demonstrated that immediate implant placement yields clinical outcomes comparable to early and delayed implantation when adequate primary stability can be achieved (Table 5).

Similarly, the available evidence suggests that immediate loading does not compromise implant survival compared with conventional loading protocols. Tealdo et al. reported no significant difference in implant survival between immediately loaded prostheses (93.9%) and conventionally loaded prostheses (95.9%) [39]. These findings support the use of immediate loading in appropriately selected patients provided sufficient primary stability is achieved.

Considerable variability exists among commercially available implant systems regarding surface characteristics, thread geometry, material composition, and implant design. However, no clinical evidence currently demonstrates superiority of a specific implant system for full-arch fixed implant-supported rehabilitation. Likewise, implant dimensions should be determined based on available bone volume, primary stability requirements, biomechanical considerations and anatomical conditions rather than manufacturer-specific characteristics [40].

A recent meta-analysis by Vouros et al. further demonstrated no significant differences in implant survival between bone-level and tissue-level implants after one and three years (relative risk 1.00 and 1.01, respectively), indicating that implant positioning relative to the crestal bone does not appear to influence short-term survival outcomes [41]. Similarly, although numerous titanium implant systems were evaluated across the included studies, no implant design generally demonstrated superior clinical performance [42]. However, direct comparisons remain limited.

Implant position and angulation constitute important biomechanical considerations in the All-on-Four concept. Posterior implants are typically tilted and positioned in the premolar region to maximize the available bone while avoiding anatomical structures. Clinical studies generally reported favourable outcomes with tilted implants, whereas experimental evidence suggests that implant angulations exceeding 30° may increase mechanical stresses and material fatigue; however, the clinical relevance of these findings remains uncertain [43]. Consequently, implant distribution and angulation should be carefully planned to optimize both biomechanical stability and long-term clinical outcomes.

Primary stability represents a prerequisite for immediate loading. According to the AWMF S3 guideline, an insertion torque between 30 and 50 Ncm is recommended before immediate loading is considered [10]. When adequate insertion torque cannot be achieved, delayed loading or placement of an alternative implant should be considered.

The number of implants also influences treatment outcomes. While the AWMF guideline recommends a minimum of four implants for fixed full-arch rehabilitation [10], comparative studies evaluating All-on-Four and All-on-Six rehabilitations have reported inconsistent findings [18,19]. Although one study observed slightly lower implant survival for All-on-Four after five years, the authors attributed this difference primarily to the limited sample size. Overall, the current evidence does not conclusively demonstrate superiority of either concept. Nevertheless, patients with sufficient posterior bone volume may benefit from placement of additional implants to improve load distribution and potentially enhance long-term implant survival.

Implant survival alone should not be considered the sole indicator of treatment success, as prosthetic complications, maintenance requirements, and patient-reported outcomes substantially influence long-term rehabilitation success.

### 4.4. Prosthetic Procedure

The use of implant-supported complete dentures has a substantial impact on prosthetic stability, function, and esthetics. Material selection plays a decisive role in long-term prosthesis performance, with both framework materials (e.g., titanium, metal alloys, zirconia) and veneering materials (e.g., PMMA, ceramic, zirconia) influencing mechanical strength, esthetic outcomes, and functional behavior.

Patients with bruxism demonstrate a significantly increased risk of prosthetic complications, particularly fractures, with reported odds ratios up to 8.7, identifying parafunctional activity as a major risk factor [9,20,22,24,28].

Notably, prosthetic failures were more frequently attributed to implant-related complications rather than material failure of the prosthesis itself. In this context, PMMA-based prostheses are considered a cost-effective interim solution due to their lower cost, ease of modification, reparability, and suitability during the adaptation phase. Therefore, they are commonly used during the provisional phase of treatment.

Acrylic veneering fractures have been associated with material fatigue induced by the magnitude, frequency, and direction of occlusal forces. Consequently, occlusal concepts such as bilaterally balanced occlusion with evenly distributed centric contacts may reduce non-axial loading and thereby decrease mechanical fatigue of restorative materials [11,26].

Maló et al. compared ceramic veneering with zirconia-based crowns and reported fewer complications in the zirconia group (16 prostheses) compared with the ceramic group (36 prostheses) [27]. In the ceramic crown group, the presence of a metal–ceramic implant-supported prosthesis in the opposing arch was identified as a significant risk factor for mechanical complications (OR = 2.04) and crown chipping (OR = 1.97). These findings suggest that zirconium dioxide crowns are preferable for patients with dentate opposing arches. Edentulous patients may benefit from prostheses with a titanium framework veneered with ceramic or zirconium dioxide for fixed implant-supported rehabilitation.

Zirconia frameworks may represent a promising option for definitive restorations; however, comparative long-term evidence remains limited. To prevent veneering ceramic chipping, monolithic reconstructions may be considered [44]. However, this approach may be rejected by patients due to aesthetic limitations, as color is applied by painting rather than layering.

Definitive prosthesis selection should consider patient preference and clinician experience. For patients with severe bruxism, a protective occlusal splint is recommended. A staged approach involving a PMMA provisional prosthesis followed by a zirconia–ceramic definitive restoration may represent a clinically reliable treatment strategy based on current available evidence.

### 4.5. Biological Complication

Marginal bone loss (MBL) is an important surrogate outcome parameter in implant dentistry, as progressive peri-implant bone loss may compromise long-term implant stability and prosthetic success. It reflects changes at the implant–bone interface and is commonly used as an indicator for monitoring peri-implant tissue health. Marginal bone loss should therefore be interpreted together with clinical parameters such as probing depth, bleeding on probing, suppuration, and prosthetic maintenance requirements. However, small differences in MBL values should be interpreted cautiously, as their clinical relevance may be limited.

Comparative studies by Toia et al. and La Monaca et al. evaluating the All-on-Four and All-on-Six concepts reported slightly higher MBL values for the All-on-Four approach after five years (0.24 mm and 1.56 mm, respectively) compared with the All-on-Six concept (0.15 mm and 1.20 mm) [18,19]. Toia et al. suggested that the relatively low MBL values observed in their study may be associated with the use of platform-switching implants, in contrast to findings reported by Tallarico et al. Nevertheless, both studies failed to demonstrate statistically significant differences between treatment concepts. Therefore, current evidence does not support the assumption that the All-on-Four approach inherently results in increased marginal bone loss compared with alternative full-arch implant configurations.

Immediate implant placement represents an integral component of the All-on-Four concept. A meta-analysis by Bassir et al., including both single implants and implant-supported full-arch rehabilitations, demonstrated slightly higher MBL values following immediate placement compared with early placement, with a mean difference of 0.14 mm favoring early implantation [45]. This difference was primarily attributed to physiological bone remodeling processes occurring within fresh extraction sockets. However, no significant differences were observed between early and delayed implant placement. Consequently, a potentially increased risk of marginal bone remodeling should be considered during treatment planning for immediate implant placement protocols, although the reported differences appear to be of limited clinical relevance.

Immediate loading is another defining characteristic of the All-on-Four concept. A meta-analysis by Benic et al. investigating single-tooth implants reported no significant differences in MBL between immediate and conventional loading protocols at two years (OR = 1.25), three years (OR = 1), and five years (1.31 mm for conventional loading versus 1.01 mm for immediate loading) [46]. Although these findings were derived primarily from single implant restorations, they suggest that immediate loading itself may not represent a major risk factor for increased marginal bone loss when appropriate implant stability and patient selection criteria are fulfilled.

Tilted implants represent another characteristic feature of the All-on-Four concept. Several studies demonstrated comparable MBL values between tilted and axially placed implants after five and ten years, respectively [12,22]. Lopes et al. reported a statistically significant difference in MBL between axial and tilted implants after five years; however, the absolute difference of 0.07 mm is unlikely to represent a clinically meaningful discrepancy [22]. Similarly, Martens et al. found no significant differences between bone-level and tissue-level implants in FDP4+ restorations after 26 months (bone-level: 1.5 ± 0.76 mm; tissue-level: 1.7 ± 0.8 mm) [30]. These findings are consistent with a meta-analysis by Vouros et al., which demonstrated only a small and statistically non-significant difference in MBL between implant designs after one year (mean difference: 0.5 mm) [41].

Peri-implant diseases, including peri-implant mucositis and peri-implantitis, represent relevant biological complications that may influence long-term treatment outcomes. These complications have been frequently reported in association with full-arch fixed implant rehabilitations, including the All-on-Four concept. Hopp et al. reported a particularly high incidence of peri-implant mucositis and suggested a higher prevalence around tilted implants [17]. However, the interpretation of these findings remains challenging due to variations in diagnostic criteria, maintenance protocols, and follow-up periods. The fixed connection between implant and prosthesis may potentially limit access for oral hygiene measures compared with removable alternatives; however, this assumption has not been sufficiently quantified in clinical studies. Therefore, further investigations are required to determine the influence of prosthetic design on long-term peri-implant health. Clinically, fixed full-arch prostheses should incorporate biologically favourable emergence profiles and allow adequate access for oral hygiene procedures. This aspect may be particularly relevant in full-arch prostheses because extensive prosthetic contours can compromise access for plaque control.

Overall, the interpretation of biological complication rates remains challenging due to substantial heterogeneity among studies, including differences in implant systems, surgical protocols, prosthetic designs, definitions of peri-implant diseases, and maintenance strategies. Future studies with standardized diagnostic criteria and long-term follow-up are required to better define risk factors associated with biological complications in full-arch implant rehabilitation.

### 4.6. Supportive Maintenance and Recall

The postoperative phase following implant placement represents a critical period for ensuring long-term stability and successful maintenance of implant-supported prostheses. Beyond the surgical intervention itself, structured supportive care and regular follow-up examinations are essential for monitoring peri-implant tissue health, prosthetic function, and the early detection of biological or mechanical complications.

Current clinical guidelines recommend individualized recall intervals based on patient-specific risk factors, prosthetic design, and oral hygiene capabilities. According to the AWMF S3 guidelines, recall intervals of three to twelve months may be considered for patients with fixed implant-supported prostheses. However, the optimal maintenance interval should be adapted according to individual risk profiles, including previous peri-implant disease, systemic conditions, smoking status, and manual dexterity. Furthermore, prosthetic treatment planning should consider the potential need for future modifications of the rehabilitation. In elderly patients or individuals who may develop age-related functional limitations, tremor, or neurological disorders, prosthetic designs that allow conversion to removable solutions may facilitate oral hygiene management and long-term maintenance [10]. Therefore, long-term treatment success depends not only on implant survival but also on the maintainability and adaptability of the prosthetic reconstruction.

### 4.7. Risk of Bias

The methodological quality of the included studies was assessed using the Newcastle–Ottawa Scale and was predominantly classified as moderate. This finding is primarily related to the observational nature of most included studies and the limited number of randomized controlled trials investigating full-arch implant rehabilitation concepts.

Several studies demonstrated comparatively lower risk of bias, particularly those with prospective study designs, clearly defined inclusion criteria, standardized outcome assessments, and adequate follow-up periods. Nevertheless, substantial heterogeneity was identified among studies regarding patient characteristics, implant protocols, prosthetic designs, outcome definitions, and follow-up durations, which may limit direct comparison and synthesis of the reported findings.

Furthermore, the predominance of non-randomized study designs introduces potential risks of selection bias and confounding. Publication bias cannot be excluded, as studies reporting favourable outcomes may be more likely to be published. These methodological limitations should be considered when interpreting reported survival rates, complication frequencies, and comparative outcomes between the All-on-Four concept and alternative full-arch rehabilitation approaches.

Overall, the available evidence provides relevant clinical information regarding the outcomes of full-arch implant-supported rehabilitations; however, the certainty of evidence remains limited due to methodological constraints and heterogeneity among studies. Future investigations should focus on standardized outcome reporting, prospective controlled study designs, and long-term follow-up to improve comparability and strengthen the evidence base.

## 5. Conclusions

Current evidence indicates that the All-on-Four concept represents a clinically established treatment option for carefully selected patients with severe posterior alveolar resorption who seek fixed implant-supported rehabilitation while avoiding extensive augmentation procedures. Implant survival and prosthetic outcomes appear comparable to alternative full-arch rehabilitation concepts; however, outcomes are strongly influenced by patient-related risk factors, anatomical conditions, implant stability, and prosthetic design.

Immediate placement and loading protocols may provide favourable outcomes when adequate primary stability and appropriate patient selection are achieved. Nevertheless, the current evidence remains limited by the predominance of observational studies, heterogeneous treatment protocols, and inconsistent outcome reporting. Therefore, further well-designed prospective comparative studies are required to establish evidence-based recommendations regarding implant configuration, loading protocols, and prosthetic materials.

Within the limitations of the available literature, the All-on-Four concept can be considered a valid treatment option for selected patients with advanced alveolar atrophy, provided that individualized risk assessment, appropriate surgical planning, and long-term maintenance are ensured.

## Figures and Tables

**Table 1:** Search strategies. * Filters.

PIO (Title/Abstract *) PubMed Results: 16	(((((((((zahnloser Kiefer[Title/Abstract]) OR (edentulous jaw[Title/Abstract])) OR (edentulous jaw[Title/Abstract])) OR (edentulous mandible[Title/Abstract])) OR (edentulous mandible[Title/Abstract])) OR (edentulous jaw[MeSH Terms])) OR (edentulous mandible[MeSH Terms])) OR (edentulous maxilla[MeSH Terms])) AND ((((((((((((Periimplantitis[Title/Abstract]) OR (Periimplantitis[Title/Abstract])) OR (Periimplantitis[Title/Abstract])) OR (Periimplantitis[Title/Abstract])) OR (chipping[Title/Abstract])) OR (chipping[Title/Abstract])) OR (chipping[Title/Abstract])) OR (biological complications[Title/Abstract])) OR (implantat survival[Title/Abstract])) OR (biological complications[MeSH Terms])) OR (biological complications[MeSH Terms])) OR (biological complications[MeSH Terms]))) AND (((((((((All-on Four System[Title/Abstract]) OR (Pro Arch[Title/Abstract])) OR (All-on 4 System[Title/Abstract])) OR (all-on-4 concept[Title/Abstract])) OR (all-on 4 surgery[Title/Abstract])) OR (all-on-four[Title/Abstract])) OR (all-on 4[Title/Abstract])) OR (four dental implants[Title/Abstract])) OR (All-on four System[MeSH Terms]))
PCO (Title/Abstract *) PubMed Results: 40	(((((((((zahnloser Kiefer[Title/Abstract]) OR (edentulous jaw[Title/Abstract])) OR (edentulous jaw[Title/Abstract])) OR (edentulous mandible[Title/Abstract])) OR (edentulous mandible[Title/Abstract])) OR (edentulous jaw[MeSH Terms])) OR (edentulous mandible[MeSH Terms])) OR (edentulous maxilla[MeSH Terms])) AND (((((((All-on six System[Title/Abstract]) OR (fixed total prosthesis[Title/Abstract])) OR (removable complete prostheses[Title/Abstract])) OR (removable complete prostheses[Title/Abstract])) OR (rehabilitation endentulous[Title/Abstract])) OR (fixed total dental prostheses[MeSH Terms])) OR (removable complete prostheses[MeSH Terms]))) AND ((((((((((((Periimplantitis[Title/Abstract]) OR (Periimplantitis[Title/Abstract])) OR (Periimplantitis[Title/Abstract])) OR (Periimplantitis[Title/Abstract])) OR (chipping[Title/Abstract])) OR (chipping[Title/Abstract])) OR (chipping[Title/Abstract])) OR (biological complications[Title/Abstract])) OR (implantat survival[Title/Abstract])) OR (biological complications[MeSH Terms])) OR (biological complications[MeSH Terms])) OR (biological complications[MeSH Terms]))
PIO (Title/Abstract *) Embase Results: 11	(‘zahnloser kiefer’:ab,ti OR edentulous:ab,ti OR ‘edentulous mandible’:ab,ti OR ‘edentulous jaw’:ab,ti) AND (‘all-on four system’:ab,ti OR ‘all-on 4 system’:ab,ti OR ‘all-on-4 concept’:ab,ti OR ‘all-on-4 surgery’:ab,ti OR ‘all on 4’:ab,ti OR ‘all on four’:ab,ti OR ‘all-on four’:ab,ti OR ‘all-on 4’:ab,ti OR ‘four dental implants’:ab,ti) AND (periimplantitis:ab,ti OR ‘peri-implant mucositis’:ab,ti OR ‘technical complications’:ab,ti OR ‘dental prosthesis fracture’:ab,ti OR ‘dental prosthesis failure’:ab,ti OR ‘biological complications’:ab,ti)
PCO (Title/Abstract *) Embase Results: 26	(((zahnloser AND kiefer OR endentulous) AND jaw OR endentulous OR edentulous) AND mandible OR edentulous) AND jaw AND ((‘all on’ AND six AND system OR fixed) AND total AND prosthesis OR removable) AND complete AND prosthesis AND (((((periimplantitis OR ‘peri implant’) AND mucositis OR technical) AND complications OR dental) AND prosthesis AND fracture OR dental) AND prosthesis AND failure OR biological)

**Table 2:** Included Studies * Studies using All-on-Four and FDP4+ methods (results for the different methods were reported separately).

	Authors	Intervention	Duration [Years]	Number of Patients	Number of Implants	Selection	Comparability	Outcome	Total Score	Risk of Bias
1	Maló et al., 2019	All-on Four	10–18	471	1884	★★★★	★	★★★	8	Low
2	Maló et al., 2019	All-on Four	5–13	1072	4288	★★★★	★	★★★	8	Low
3	Grandi ad Signorini 2021	All-on Four	10	96	384	★★★★	★	★★★	8	Low
4	La Monaca et al., 2022	All-on Four	10	28	68	★★★★	★	★★	7	Low
5	Lopes et al., 2015	All-on Four	7	23	92	★★★★	★	★★	6	Moderate
6	Nobre et al., 2025	All-on Four	20–25	181	724	★★★★	★	★★	7	Low
7	Lopes et al., 2017	All-on Four	7	111	1532	★★★★	★	★★★	8	Low
8	Li et al., 2017	All-on Four	5 (2–7)	17	80	★★★	★	★★	6	Moderate
9	Cavalli et al., 2016	4 implants	1–10	43	196	★★★	★	★★	6	Moderate
10	Hopp et al., 2017	All-on Four	5	891	3564	★★★★	★★	★★	9	Low
11	Tallarico et al., 2016	All-on Four	7	56	224	★★★★	★	★★	7	Low
*	Toia et al., 2024	All-on Four	5	28	112	★★★	★★	★★	7	Low
1	Toia et al., 2024	All-on Six	5	28	168	★★★	★★	★★	7	Low
2	Agliardi et al., 2021	All-on Four hybrid (zygomatic) Standard implants	11	34	90 zygomatic 53 standard	★★★	★	★★★	7	Low
3	Schwarz et al., 2010	>4 implants Early loading	4.5 (1–8)	37	185	★★★	★	★★	6	Moderate
4	Ekelund et al., 2003	>4 implants	>20	47	273	★★★	★	★★	6	Moderate
5	Maló et al., 2012	≥4 implants	10	108	634	★★★★	★	★★★	8	Low
6	Maló et al., 2011	≥4 implants different resorption	5	221	995	★★★	★	★★	6	Moderate
7	Velasco-Ortega et al., 2021	>8 implants Immediat implant loading	7	22	198	★★★	★	★★	6	Moderate
8	Martens et al., 2014	>4 implants Immediat loading	5	33	163	★★★★	★	★★	7	Low
9	Fischer et al., 2008	>4 implants Early and delayed implant laoding	5	24	142	★★★	★	★★	6	Moderate
*	La Monaca et al., 2022	All-on Six	10	28	96	★★★★	★	★★	7	Low

**Table 3:** Excluded study with reason.

	Excluded Study	Reason for Exclusion
1	Maló et al., 2013	Only zygomatic implants
2	Tironi et al., 2023	Not fully access
3	Wong et al., 2015	Only zygomatic implants
4	Peñarrocha-Oltra et al., 2013	Not enough information

**Table 4:** Summary of study parameters and outcomes.

Patient	Indication	Patients requiring full-arch restoration through immediate function with a complete edentulous mandible or maxilla, good physical and psychological health
	Contraindication	Heavy smokers (>15 cigarettes/day), pregnancy or lactation, lack of compliance, limited bone volume, metabolic bone disease or bisphosphonate therapy history, head and neck region radiotherapy or chemotherapy history
	Bone	Bone volume up to first and second premolar in volume and density >4 mm wide horizontally and >8 mm high
Surgical Procedure	Implant Insertion	Guided implant insertion with ≥30 Ncm, immediately, early and delayed loading depending on the surgeon and patient
	Implant Type	Preferred by the surgeon, height (≥7 mm) and diameter (≥3.5 mm) according to the bone supply, axial or tilted (30–45°), bone level and tissue level
Prosthodontic Procedure	Temporary Prostheses	Patients request or PMMA
	Final Prostheses	Patients request, Metal/Zirconia—framework with PMMA or ceramic

**Table 5:** Implant design and implant survival rate in the All-on-Four concept and (fixed implant-supported prostheses with more than four implants) FPD4+ I_S = Implant s vival. (†) Self-tapping implant. (1) Bone-level implant. (2) Tissue-level implant. N/A = Not Available, * Studies using All-on-4 and FDP4+ methods (results for the different methods were reported separately).

	Authors	Intervention	Type of Implants	Implant Diameter [mm]	Implant Length [mm]	Distal Tilted	Immediate Implantation	Immediate Loading	I_S 1. Year	I_S 5. Year	I_S 7. Year	I_S ≥10 Years
1	Maló et al., 2019	All-on Four	Brånemark System Mk II, Mk III, Mk IV ^2^ NobelSpeedy ^†1^	3.75	>7	Yes	Yes	Yes	99.60%	98.50%	98.30%	96.90% (10) 93% (18)
2	Maló et al., 2019	All-on Four	Brånemark-System MK III/IV ^2^	N/A	N/A	Yes	Yes	Yes	98.60%	97.70%	97.40%	96.40% (10) 94.70% (13)
3	Grandi ad Signorini 2021	All-on Four	JDEvolution ^†^	3.2–5	10–15	Yes	Yes	Yes	N/A	N/A	N/A	97.90% (10)
4	La Monaca et al., 2022	All-on Four	NobelSpeedy Groovy ^†1^	3.3–4	7–18	Yes	Yes	Yes	N/A	N/A	N/A	89.70% (10)
5	Lopes et al., 2015	All-on Four	NobelSpeedy Groovy ^†1^	4	8–18	Yes	Yes	Yes	97.80%	96.60%	96.60%	N/A
6	Nobre et al., 2025	All-on Four	NobelSpeedy Groovy ^†1^	4	8–18	Yes	Yes	Yes	99%	98.1%	97.7%	95.9% (10) 90% (25)
7	Lopes et al., 2017	All-on Four	Brånemark System Mk II, Mk III, Mk IV ^2^ NobelSpeedy ^†1^	N/A	10–18	Yes	Yes	Yes	96.30%	94.50%	94.50%	N/A
8	Li et al., 2017	All-on Four	Nobel Speedy ^†1^ Brånemark System MK III ^2^ NobelActiv ^†^	3.5–3.75	10–18	Yes	Yes	Yes	N/A	98.75%	N/A	N/A
9	Cavalli et al., 2016	4 implants	NobelSpeedy Groovy ^†^ Brånemark System, MKIV ^2^	N/A	N/A	Yes	Yes	Yes	100%	100%	100%	100% (10)
10	Hopp et al.,, 2017	All-on Four	NobelSpeedy Groovy ^†^ Brånemark System MKIII, MKIV ^2^	3.3–5	7–18	Yes	Yes	Yes	N/A	95.7% (axial) 96.1% (tilted)	N/A	N/A
11	Tallarico et al., 2016	All-on Four	NobelSpeedy Groovy ^†1^ NobelReplace Tapered Groovy ^†1^ Brånemark System MKIII ^2^	N/A	N/A	Yes	Partly	Yes	99.60%	99.60%	99.60%	N/A
*	Toia et al., 2024	All-on Four	OsseoSpeed TX ^1^	N/A	N/A	Partly	N/A	No	100%	99.3%	N/A	N/A
1	Toia et al., 2024	All-on Six	OsseoSpeed TX ^1^	N/A	N/A	Partly	N/A	No	100%	100%	N/A	N/A
2	Agliardi et al., 2021	All-on Four hybrid (zygomatic) Standard implants	NobelZygoma	N/A	N/A	Partly	N/A	N/A	100% 98.10%	100% 96.20%	100% 96.20%	N/A
3	Schwarz et al., 2010	>4 implants Early loading	FRIALOC ^1^	3.5–4	10–13	N/A	No	No	N/A	89.70%	N/A	N/A
4	Ekelund et al., 2003	>4 implants	Brånemark MK System ^2^	N/A	10	N/A	N/A	No	N/A	99.30%	N/A	98.90% (10) 98.90% (20)
5	Maló et al., 2012	≥4 implants	Branemark system, ^2^ Nobel Speedy ^†1^	N/A	N/A	Yes	Yes	Yes	N/A	N/A	N/A	N/A
6	Maló et al., 2011	≥4 implants different re sorption	Brånemark MKII, MKIII, MKIV ^2^ NobelSpeedy ^†1^	3.3–4	10–18	Yes	Yes	Yes	97.40%	95.80%	N/A	N/A
7	Velasco-Ortega et al., 2021	>8 implants Immediate implant loading	N/A	3.5–4	10–12	N/A	Yes	Yes	N/A	N/A	97.50%	N/A
8	Martens et al., 2014	>4 implants Early and delayed implant loading	Biomet 3i NanoTite ^1^/Osseotite ^2^	3.25 (1) / 4	10–15	N/A	Yes	Yes	96.30%	94.40%	N/A	N/A
9	Fischer et al., 2008	>4 implants Early and delayed implant loading	Straumann Standard Plus Tissue level ^2^	4.1	8–12	N/A	No	N/A	97.90%	95.10%	N/A	N/A
*	La Monaca et al., 2022	All-on Six	NobelSpeedy Groovy ^†1^	3.3–5.5	7–13	Yes	Yes	Yes	N/A	N/A	N/A	99.00% (10)

**Table 6:** Prosthetic material for fixed implant-supported oral prostheses with their survival rate. * Only 2 authors describe the survival rate of temporary prostheses. N/A = Not Available.

	Autors All-on-Four	Autors with an Other FDP-Intervention	Mechanical Complications	Survival All-on Four (5–18 Years)	Survival Other FDP-Intervention (3–20 Years)
**Temporary Prostheses**					
PMMA	Maló et al., 2019; Maló et al., 2019; Grandi and Signorini 2021; La Monaca et al., 2022; Lopes et al., 2015; Nobre et al., 2025; Lopes et al., 2017; Li et al., 2017; Cavalli et al., 2016.	Monaca et al., 2022b; Schwarz et al., 2020; Velasco-Ortega et al., 2021	9.10-81.90%	85.00–100%	100% *
PMMA with titanium cylinders	Hopp et al., 2017	Maló et al., 2011	N/A		
PMMA with composite teeth		Agliardi et al., 2021	N/A		
Metal-reinforced with PMMA	Tallarico et al., 2016		N/A		
**Final Prostheses**					
Metal Framework with PMMA	Lopes et al., 2017; Cavelli et al., 2016; Toia et al., 2024; Lopes et al., 2015;	Toia et al., 2024; Schwarz et al., 2020	7.30–36.70%	88.20–100%	89.20–100%
Metal Framework with ceramic	Maló et al., 2012; Tallarico et al., 2016; Hopp et al., 2017b; Li et al., 2017; Lopes et al., 2015;	Maló et al., 2011; Maló et al., 2012a; Velasco-Ortega et al., 2021	7.27–18.20%		
Metal Framework with zirconia	Nobre et al., 2014, Lopes et al., 2017		54.7%		
Zirconia with ceramic	Tallarico et al., 2016		N/A		

**Table 7:** Biological complications of the All-on-Four concept compared to other implant-supported restorations (1) abscess, * Studies using All-on-4 and FDP4+ methods (results for the different methods were reported separately), ** recorded by event, not by patient.

MBL	Authors	Intervention	Number of Patients	MBL 1. Jahr [mm]	MBL 3. Jahr [mm]	MBL 5. Jahr [mm]	MBL ≥7 Jahre [mm]	Implant Infection [Number of Patients]	Gingiva inflamation [Number of Patients]	Perimplant Pathologies [Number of Patients]
1	Maló et al., 2019	All-on Four	471	N/A	N/A	N/A	1.98 (10) 2.23 (15)	N/A	12	108
2	Maló et al., 2019	All-on Four	1072	N/A	N/A	1.18	1.67 (10)	203	N/A	106
3	Grandi ad Signorini 2021	All-on Four	96	N/A	N/A	1.50	1.8 (7) 2.5 (10)	13	N/A	N/A
4	La Monaca et al., 2022	All-on Four	28	N/A	N/A	1.56	N/A	2	0	0
5	Lopes et al., 2015	All-on Four	23	1.70	1.70	1.90	N/A	N/A	N/A	2
6	Nobre et al., 2025	All-on Four	181	N/A	N/A	N/A	2.28 (25)	N/A **	N/A **	N/A **
7	Lopes et al., 2017	All-on Four	324	N/A	N/A	1.74 (axial) 1.76 (tilted)	N/A	N/A	N/A	N/A
8	Li et al., 2017	All-on Four	17	0.8 (axial) 0.9 (tilted)	0.9 (axial) 0.9 (tilted)	1.0 (axial) 1.1 (tilted)	1.2 (axial) 1.2 (tilted)	1	N/A	N/A
9	Cavalli et al., 2016	4 implants	43	N/A	N/A	N/A	N/A	1	1	N/A
10	Hopp et al.,, 2017	All-on Four	891	N/A	N/A	1.14 (axial) 1.19 (tilted)	N/A	24	189	95
11	Tallarico et al., 2016	All-on Four	56	0.97	N/A	N/A	1.3	3	1	N/A
*	Toia et al., 2024	All-on Four	28	0.2	0.22	0.24	N/A	0	3	1
1	Toia et al., 2024	All-on Six	28	0.15	0.12	0.15	N/A	1 ^1^	5	4
2	Agliardi et al., 2021	Zygomatic standard implants	34	0.85	1.10	1.18	1.36 (10)	N/A	N/A	0
3	Schwarz et al., 2020	>4 implants Early loading	35	N/A	N/A	N/A	N/A	3	N/A	N/A
4	Ekelund et al., 2003	>4 implants	47	N/A	N/A	N/A	1.4 (15) 1.6 (20)	N/A	6	N/A
5	Maló et al., 2012	Prosthetic material	108	N/A	N/A	N/A	N/A	9	N/A	7
6	Maló et al., 2011	≥4 implants different resorption	221	N/A	N/A	N/A	N/A	N/A	N/A	N/A
7	Velasco-Ortega et al., 2021	>8 implants Immediate implant loading	22	N/A	N/A	N/A	1.44	10	N/A	N/A
8	Martens et al.,	>4 implants Immediate loading	33	N/A	1.5 (2J)	1.60	N/A	N/A	N/A	N/A
9	Fischer et al., 2008	>4 implants Early and delayed implant loading	24	2.5 (early) 3.3 (delayed)	2.7 (early) 3.5 (delayed)	2.9 (early) 3.7 (delayed)	N/A	N/A	N/A	N/A
*	La Monaca et al., 2022	All-on Four All-on Six	28	N/A	N/A	1.20	N/A	0	1	0

## Data Availability

All data analyzed in this systematic review are derived from previously published studies and are included in this article. No new data were generated.

## Supplementary Materials

The following supporting information can be downloaded at: https://www.mdpi.com/article/10.3390/dj14090541/s1, Table S1: PRISMA 2020 Checklist.

## Abbreviations

The following abbreviations are used in this manuscript:

AWMF	Association of Scientific Medical Societies
FDP4+	Fixes dental prostheses with more than four implants
MBL	Marginal bone loss
OR	Odds ratio
PMMA	Polymethyl methacrylate/acrylic resin
Tooth 32, 42	Second incisor in the lower jaw; FDI notion
Tooth 35, 45	Second premolar in the lower jaw; FDI notion
